# Psilocybin-assisted psychotherapy for methamphetamine dependence: a case report involving daily methamphetamine use

**DOI:** 10.3389/fpsyt.2024.1490907

**Published:** 2024-12-06

**Authors:** Jonathan Brett, Elizabeth Knock, Kathy Watson, Steven Albert, Krista J. Siefried, Jeffrey Guss

**Affiliations:** ^1^ St. Vincent’s Clinical School, University of New South Wales, Sydney, NSW, Australia; ^2^ School of Population Health, University of New South Wales, Sydney, NSW, Australia; ^3^ St. Vincent’s Hospital Alcohol and Drug Service, St. Vincent’s Hospital, Sydney, NSW, Australia; ^4^ School of Psychiatry, Faculty of Medicine, University of New South Wales, Sydney, NSW, Australia; ^5^ The National Centre for Clinical Research on Emerging Drugs of Concern (NCCRED), c/o The University of New South Wales, Sydney, NSW, Australia; ^6^ National Drug and Alcohol Research Centre (NDARC), The University of New South Wales, Sydney, NSW, Australia; ^7^ New South Wales Drug and Alcohol Clinical Research and Improvement Network (DACRIN), c/o the Ministry of Health, Sydney, NSW, Australia; ^8^ New York University (NYU) Postdoctoral Program in Psychotherapy and Psychoanalysis, New York University (NYU) Grossman School of Medicine, New York, NY, United States

**Keywords:** psilocybin, psychotherapy, methamphetamine use disorder, treatment, psychedelic, methamphetamine dependence

## Abstract

Methamphetamine (MA) dependence leads to severe physical and psychological issues. Current treatments, including psychosocial therapies and residential rehabilitation, face limitations such as high relapse rates, cost, and accessibility issues. As a result, there is an urgent need for novel approaches to treat MA dependence that are effective, affordable, and accessible to patients. Psilocybin, the active component in numerous mushrooms of the *Psilocybe* genus, has shown potential for enhancing psychotherapy for various addiction and mental health issues due to its effects on perception, cognition, and affect. Psilocybin-assisted psychotherapy (PAT) has demonstrated initial safety and efficacy in treating alcohol, cocaine, and nicotine dependence. The case presented here describes a 36-year-old transwoman and daily MA user, who participated in a single-arm open-label clinical trial assessing feasibility and safety of PAT for MA dependence at St. Vincent’s Hospital, Sydney. Following inpatient withdrawal management and one session of psilocybin-assisted therapy, she experienced significant cognitive and emotional shifts and sustained MA abstinence. She reported improved mental health over 3 months following treatment completion. She also noted increased self-esteem, mindfulness, and distress tolerance. This study suggests that PAT (following inpatient MA withdrawal management) may offer a scalable, safe, and effective approach for treating MA dependence. However, further research is required to confirm the generalisability and efficacy of PAT for broader populations of people using MA. It is encouraging that this participant, a daily MA user, showed improvements in mood and cognition, in addition to abstinence from MA.

## Introduction

The global prevalence of amphetamine (including methamphetamine) use is estimated to be 0.7%, with 11% of those actively using at any given point in time meeting criteria for dependence (1). There are concerns that stimulant dependence is increasing globally because of changes in the global markets of other drugs, including heroin (2). Methamphetamine (MA) dependence is associated with a range of short- and long-term adverse effects, including depressive symptoms, anxiety, psychosis, cardiovascular diseases, and increased risk of HIV and hepatitis C infection (1). Compared to those not dependent, people with methamphetamine dependence have almost seven times greater mortality (1). MA use disproportionately impacts intersectional priority populations including Indigenous People, adolescents, and LGBTQ+ populations (3–5). There are no robustly effective pharmacotherapies available (6), and other clinically available treatments, including long-stay residential rehabilitation, carry substantial constraints related to access, cost, and scalability (7). Specific psychosocial therapies such as cognitive behavioural therapy have demonstrated weak effects, high relapse rates (8), and poor consumer acceptability (9). Contingency-management-type treatments have proven costly but are effective in improving treatment retention, abstinence, and frequency of methamphetamine use. However, clinical use of CM has been limited (10), and so there is a dire need to find novel and effective approaches to treat MA dependence.

Psilocybin is a serotonin 5-HT_2A_ receptor agonist with very low abuse potential (11). It produces acute and transitory changes to perception, cognition, and affect. Critically, psilocybin, when delivered as an adjunct to addiction-focused psychotherapy, has demonstrated the capacity to safely enhance affective and cognitive impacts of such treatments (12). Psilocybin-assisted psychotherapy (PAT) has been studied as a treatment for a range of mental health and addiction concerns (12). Specifically, psilocybin-assisted psychotherapy has demonstrated safety and efficacy in phase II trials of alcohol dependence (13). There is emerging evidence from early phase trials of nicotine dependence (14) and a recently completed phase II trial of cocaine dependence. There is strong rationale for investigating psilocybin-assisted psychotherapy as a novel treatment for MA dependence (15).

Here, we offer a clinical case summary of one participant of a study of psilocybin-assisted psychotherapy for methamphetamine dependence conducted at St. Vincent’s Hospital (Sydney, Australia). We aim to illustrate how this intervention can be delivered to an individual with daily methamphetamine use and psychosocial complexity.

## Study methods

The case described below was part of a single-arm, open-label safety, and feasibility study conducted in an outpatient setting (ACTRN12622000463774) in which participants seeking treatment for methamphetamine dependence were recruited from a community hospital MA addiction treatment program involving outpatient psychotherapy. Eligibility criteria included engagement with a non-study therapist prior to enrolment so that ongoing support could be provided following study conclusion, presence of a supportive living environment, and ability to maintain abstinence from MA for at least 2 days prior to psilocybin dosing without features of severe withdrawal. The latter was assessed by a medical officer prior to psilocybin dosing with the assistance of the Amphetamine Withdrawal Scale (16) and urine drug analysis. Participants were excluded if they had a personal or family history (first- or second-degree relatives) of psychosis or bipolar disorder, history of seizure disorder, or uncontrolled hypertension or use of any psychedelic within the month prior to recruitment. Additionally, participants had to have stable cardiopulmonary, renal, hepatic, and cognitive functioning as assessed by a consultant physician with a medical history, physical and mental state examination, electrocardiogram, and blood tests.

The intervention included three preparatory psychotherapy sessions over the course of 2 weeks, each lasting approximately 90 min. These sessions aimed to establish therapeutic alliance, familiarise the participant with the treatment setting, provide psychoeducation, explore the participant’s life circumstances and history, clarify participant intentions for treatment, and support coping strategies to navigate potentially challenging experiences.

Following the three preparatory sessions, participants were administered a dose of 25 mg oral psilocybin, followed by 6 h of unobtrusive, supportive care. The dosing session took place in a living-room-like environment within the hospital. During this time, participants were invited to lie on a sofa, wear an eye mask, and listen to a curated music playlist in the presence of the two therapists who had conducted the preparatory sessions. The therapists offered supportive therapy as required, including judicious use of touch for support. The use of touch was agreed upon during preparation psychotherapy and could only occur below the elbow during psilocybin dosing. Following assessment of stability for leaving the research setting, at the end of the day, participants were released to home, accompanied by a family member or support person.

Participants were given two further 90-min integration psychotherapy sessions: the first occurred 1 day following the dosing session, and the next session occurred approximately 1 week later. These sessions focused on describing and exploring participant’s experiences during the dosing sessions and making links and connections with intentions and motivations for change developed during preparation sessions. Following these two integration sessions, participants were linked back to “care as usual” in the MA treatment program from which they were recruited.

All psychotherapy was administered by a consistent therapeutic dyad, consisting of two licensed therapists. These therapists followed a detailed guide incorporating principles of motivational interviewing and acceptance and commitment therapy (ACT) and informed by published manuals used in prior successful studies of psilocybin-assisted psychotherapy [35, 36].

Participants were followed weekly for 1 month and then finally at 3 months following the psilocybin dose to measure safety and tolerability; change in methamphetamine use over the prior 28 days (17); MA cravings; depression, anxiety, and stress ratings (18); and quality of life (19). Participants also underwent two 30–60-min interviews with an independent qualitative researcher; the first occurred prior to initiating psychotherapy and the second approximately 1 month following psilocybin dosing. Interviews explored motivations to participate in the study and experiences of the study intervention.

The study was approved by St. Vincent’s Human Research Ethics Committee (2020ETH01695), and PM (pseudonym) gave written and verbal consent to have her case written up as a case report; she reviewed and approved the final version offered here.

## Case presentation

### Participant history

PM is a 36-year-old transwoman (preferred pronouns she/her) who first used MA in the context of recreational sexual activity 2 years prior to study enrolment. Sexual encounters were typically not with a regular partner. Her initial motivations for MA use were to enhance energy and sexual appetite. Her use of MA progressed to daily over the following year and was not always linked to sexual activity. Her MA consumption increased from 0.1 g to 0.5 g per session over a similar period (the year prior to entry into the study). PM described typical stimulant withdrawal symptoms following MA abstinence including 2–3days of extreme lethargy followed by increased craving for MA. PM smoked MA and denied IV use. Concomitant substance use included gamma hydroxybutyrate (GHB) in the context of sex once or twice per week over the prior year and daily vaping of nicotine. She denied other recent substance use. PM reported one psychedelic experience with LSD 10 years prior to entering the current study. This occurred in a party setting and was described as a negative experience. She became aware of the current study through a friend who had been a participant with a positive outcome and on that basis was motivated towards participating.

PM had been treated by a psychotherapist fortnightly for the 3 years prior to study enrolment. Twelve months prior to the study, she had had 2 months of full abstinence but returned to daily use 6 months prior to study enrolment and had since been unable to reduce her MA use. Her main concerns regarding her MA use included constant exhaustion and fatigue both when using and not using, difficulties forming and maintaining relationships with those not using, and frequently dosing beyond her intended duration. She also described ruminative/obsessional preoccupation and loss of time that was devoted to deep focus on non-urgent reading or conversations and meaningless manual tasks (she described these as “cractivities”). These adverse consequences of MA use impaired her achieving her life goals, which centred around a career in the arts.

PM described her childhood as mostly happy and denied major traumatic experiences. She described her relationship with her parents as loving and supportive and has two supportive sisters. She completed high school, studied performing arts at university, and worked in theatre in a range of roles. Six months prior to study enrolment, she had started engaging in sex work, which she found both lucrative and mostly enjoyable. She felt compelled to use methamphetamine to boost stamina in longer sex sessions and/or to be on “the same level” as clients. She also described MA as a shortcut to intimacy in both sex work and her personal sexual experiences.

She began identifying as genderqueer (a non-binary gender identity) in her early 20s and had shifted her physical presentation towards a more femme aesthetic incrementally over time. She asserted a transgender and transfeminine identity and started hormone replacement therapy at 33 years old. At the time of the study, she was part way through a 2+ year course of electrolysis to remove facial hair but had no other surgeries. She is aware of an ongoing non-urgent desire to undertake small amounts of facial feminisation surgery including a tracheal shave.

Since her social gender transition, she had one committed relationship lasting 6 months and numerous overlapping ongoing sexual relationships. At baseline assessment, she described being in a committed but tenuous relationship with a man was also methamphetamine dependent. She described having a supportive network of friends, a combination of people who did and did not use methamphetamine.

She began having mental health difficulties in high school and experienced some depressive symptoms in adolescence associated with gender and sexual identity issues. Symptomatology included passive suicidal ideation for which she did not seek treatment. In her 30s, she had experienced periods of low mood and was diagnosed with Attention Deficit Hyperactivity Disorder (ADHD) by a psychiatrist 1 year prior to entering the current study. However, given a stipulation of 12 months MA abstinence confirmed by regular urine drug screening before she could be prescribed psychostimulants, PM preferred to self-medicate with methamphetamine and attempt to self-manage her ADHD by building coping mechanisms for focus and attention rather than undergo such a level of surveillance and thus disengaged with treatment. While methamphetamine helped with focus and returning to a “sense of normal,” she acknowledged the adverse effects of this approach. Her prescribed medication at the time of enrolment included gender-affirming hormonal medications including 6 monthly oestradiol pellet implants and daily spironolactone and Truvada for HIV pre-exposure prophylaxis.

She presented as an intelligent and articulate person who was frustrated with the way MA interfered with her ability to think clearly. At screening, she described using MA during 27 of the prior 28 days.

### Study treatment

#### Preparatory therapy

PM completed two preparatory psychotherapy sessions over the course of 5 days during which she was neither intoxicated or in withdrawal and able to engage with therapy. She was admitted for inpatient withdrawal management immediately following the second session. PM remained an inpatient for withdrawal management for 5 days; the third preparatory session occurred on day 4 of admission. PM received a dose of psilocybin the following morning and was then discharged into the community once she was deemed stable enough to leave the research setting.

During inpatient withdrawal management, PM received low-dose diazepam (5–10 mg per day orally) for the first 2–3 days of admission but no pharmacotherapy in the 48 h prior to psilocybin dosing. She described this period as challenging but necessary. On the morning of the psilocybin dosing session, she was discharged from the inpatient unit to the Psychedelic Assisted Therapy (PAT) clinic where she was assessed to not have features of MA intoxication or withdrawal and then took the 25 mg psilocybin orally.

PM was quiet for approximately 1 h, following her dose, and then began to show clenching and writhing of her limbs during the second hour. Following this, she demonstrated restlessness and mild agitation, moving around on the couch for the next few hours, and remained internally focused for extended periods of time. There were moments of visible emotional expression, with crying, and laughter, and verbal expressions of joy and awe. When she stood to go to the bathroom, she spoke briefly with the therapists. After returning to the couch, she returned to use of the headphones and eyeshade. Her vital signs remained normal throughout the day. During subsequent self-assessment, she scored >60% on each of the seven subscales of the mystical experience questionnaire, indicating a “complete mystical experience” (20) and scored a total of 80 out of 100 on the emotional break through inventory (21). Following resolution of her psilocybin effects, she was discharged home into the care of a friend.

She returned for a session the following day, then returned 1 week later for integration psychotherapy. During these therapy sessions, she discussed her psilocybin-assisted therapy experience—including transcendent and challenging content and its impact on her MA use. During her psychedelic experience, she described an ability to see her life to date in “great distance and perspective” to see the “ecology” of her life, the “interconnectedness” of events leading up to the present and “relationship with amphetamine … with family and friends and professional pursuits.” She also reported a series of insights that occurred in the days following the dosing session that led to positive effects on self-esteem and mindfulness. She described the “capacity to make … changes swiftly” and observed a sense of equanimity in herself, in the “idea that pain or grief or suffering is a sensation in the body that is almost exactly the same as joy or fulfilment or loving kindness.” She also described increasing distress tolerance by “watching [difficult experiences] move through [her] body and pass out of [her] body” and through this understood “how not to avoid those things but to go through them.”

During the independent interview conducted 2 months following dosing, she reflected on the importance of “specialist” therapist support throughout the intervention to identify therapeutic goals that were distinct from therapy with her usual therapist. She engaged actively in planning alternate activities to enhance her sober experiences of creativity and social connection. Despite her initial goal being to reduce MA use, she shifted to complete abstinence following PAT. During follow-up visits, she denied any subsequent use of MA, up to 3 months following the dosing day that was verified by drug urinalysis. Her MA cravings as measured by a visual analogue scale (0–100) were 100 at baseline and 28 out of 100 on day 28 and 41/100 on day 90 post-dose. Her Depression, Anxiety, and Stress (DASS-21) score was 13 out of 21 at baseline and 6/21 and 7/21 at days 28 and 90. Her quality of life measured by the WEMBS was 54 out of 70 at baseline, 57/70 on day 28 and 50/70 on day 90. During this time, PM separated from her partner, which she associated with her abstinence from MA. She attributed increased craving at 3 months to stressful life circumstances and reflected that, despite ongoing support from her regular community therapist, she would have found an additional integration session with her PAT therapists valuable, should one have been offered 2–3 months following treatment session. PM experienced no adverse effects during the trial but admitted to “microdosing” with small amounts of mushrooms approximately 3 months following her initial dose on less than five occasions with the intent of achieving “neuro-lucidity to understand the whole mess” and expressed an interest in having another microdosing experience but in a therapeutic context if this were possible.

## Discussion

This case in the context of a clinical trial demonstrates promise for an innovative method of treatment for MA dependence. The individual described in this case report demonstrated cessation of MA and improvements in mental health symptoms following treatment.

MA dependence is a complex condition, characterised by symptoms that manifest physically, mentally, and emotionally (1). Pharmacotherapy alone and psychosocial therapy alone have shown poor results in terms of lasting treatment effects (6, 8). This case report suggests that the ability to identify treatment goals, and work towards integrating changes into daily living, may be enhanced in psilocybin-assisted therapy (PAT), perhaps in part due to the effects of psilocybin on enhancing neuroplasticity and motivation to change (15). In prior surveys, most US addiction specialists and psychiatrists surveyed appeared optimistic about the possibility of psychedelics being used to treat addictions. However, there was more optimism for the treatment of other psychiatric conditions. Additionally, concerns around the addictive potential of psychedelics was identified as a predictor of psychiatrists not believing that psychedelics hold potential for treatment substance use disorders (22, 23), despite evidence that the abuse potential of psychedelics is negligible (11) and growing evidence of the efficacy and safety of PAT to treat substance use disorders (24). Our study is the first to investigate the use of PAT to treat methamphetamine dependence. While PM did use small amounts of psilocybin containing mushrooms following the trial, this was with therapeutic intent, and had clinical options been available, PM states that this would have been preferable.

As seen in this case, PM identified a series of positive effects on MA use following PAT. Prior to the study, her use became problematic; she was unable to cease MA use and maintain abstinence beyond 2 months. She found that renewed recreational use slid quickly back towards daily use, particularly exacerbated by periods of stress and overwork. Her experience of PAT brought a new perspective and self-efficacy and insights into the series of life events connected to her addiction. Following integration therapy, she experienced improvements in self-esteem and distress tolerance. This manifested in major life changes, including the ability to separate from her partner, with whom she used MA. Despite these psychosocial challenges, her renewed capacity to sit with distress helped her remain abstinent from MA through this period.

Much remains to be understood about PAT, and whether the changes seen in this participant are attributable to her experience of this study cannot be known. However, given her motivation to change (i.e., voluntarily seeking participation in this study), we can posit that the neural and behavioural plasticity brought about in PAT allowed her to identify goals for herself and work towards achieving those treatment goals.

Psychosocial therapies for MA dependence generally do not require withdrawal management prior to treatment initiation in most community settings (8). Similarly, most trials of pharmacotherapies for MA dependence to date have not required withdrawal management or MA abstinence prior to study enrolment (6, 25). Hence, the role of withdrawal management in ongoing community MA treatment is unclear. The impact of inpatient withdrawal on treatment outcomes is unknown (25). In contrast, residential rehabilitation settings either require withdrawal management prior to entry or provide this as part of treatment on admission. According to our study protocol, PAT is positioned somewhere in between. Eligibility for our study included at least 2 days of abstinence from MA use and the absence of features of intoxication or acute withdrawal after this period and prior to psilocybin dosing. These requirements were selected to mitigate risks of serotonin or sympathomimetic toxicity and a negative or attenuated psychedelic experience that might result from drug interactions or cognitive alterations associated with intoxication or withdrawal states. Consistent with this, in their study of PAT for alcohol dependence, Bogenschutz et al. also avoided administering psilocybin to participants in active alcohol withdrawal (13). In Australia, there are more individuals who use MA on a “weekly to monthly” basis than those who use daily (26). The ability of this population to refrain from MA use and maintain periods of abstinence for several days at a time lends itself to PAT. However, the greatest harms of MA are experienced by the population who use MA daily or almost daily, and so this population has the most urgent treatment need. PAT for methamphetamine dependence is in its nascence.

## Conclusion

While the case presented here describes how PAT can be safely and feasibly administered to a person using MA daily by integrating PAT with MA withdrawal management, more research is required to determine how to bring this treatment to a broader range of individuals seeking treatment for MA dependence. Larger randomised controlled studies (RCTs) are required to further study PAT and its potential as a safe and effective treatment of MA dependence.

## Data Availability

The original contributions presented in the study are included in the article/supplementary material, further inquiries can be directed to the corresponding author/s.
